# 

**Published:** 1862-04

**Authors:** 


### CONTENTS.

ORIGINAL ESSAYS AND COMMUNICATIONS.

Smells, and their Management. By Dr. Geo. Watt, ........ 149
Atmospheric Pressure. By Dr. W. H. Atkinson, ........ 158
New Applications of Vulcanized Rubber. By Dr. J. Richardson, ........ 161
Warranting Dental Operations. By J. Taft, ........ 168

SELECTIONS.

Causes of Professional Ignorance, ........ 172
Death from Chloroform at the Bellevue Hospital, New York, ........ 175
Reading in the Dental Profession, ........ 179
Vulcanite Work ........ 184

CORRESPONDENCE.

Letter from Philadelphia, ........ 185

PROCEEDINGS OF SOCIETIES.

Mad River Valley Dental Society, ........ 188

EDITORIAL.

Articulating Teeth on Rubber Work ........ 188
The Uses of Rubber, ........ 190
"Nursing her Wrath to keep it Warm," ........ 190
Notice of Correspondence ........ 191
Directions for Vulcanite Work, ........ 192
Amalgam in England, ........ 194
"A Agriement Made and Enterd," ........ 195
Dental Supplies, ........ 195
Dental Depot, ........ 196
Dental Practice, ........ 196

